# Computer vision supported pedestrian tracking: A demonstration on trail bridges in rural Rwanda

**DOI:** 10.1371/journal.pone.0241379

**Published:** 2020-10-26

**Authors:** Evan Thomas, Sally Gerster, Lambert Mugabo, Huguens Jean, Tim Oates

**Affiliations:** 1 Mortenson Center in Global Engineering, University of Colorado Boulder, Boulder, Colorado, United States of America; 2 Amazi Yego Ltd., Kigali, Rwanda; 3 Synaptiq Inc., Portland, Oregon, United States of America; 4 Department of Computer Science and Electrical Engineering, University of Maryland Baltimore County, Baltimore, Maryland, United States of America; University Tunku Abdul Rahman, MALAYSIA

## Abstract

Trail bridges can improve access to critical services such as health care, schools, and markets. In order to evaluate the impact of trail bridges in rural Rwanda, it is helpful to objectively know how and when they are being used. In this study, we deployed motion-activated digital cameras across several trail bridges installed by the non-profit Bridges to Prosperity. We conducted and validated manual counting of bridge use to establish a ground truth. We adapted an open source computer vision algorithm to identify and count bridge use reflected in the digital images. We found a reliable correlation with less than 3% error bias of bridge crossings per hour between manual counting and those sites at which the cameras logged short video clips. We applied this algorithm across 186 total days of observation at four sites in fall 2019, and observed a total of 33,800 daily bridge crossings ranging from about 20 to over 1,100 individual uses per day, with no apparent correlation between daily or total weekly rainfall and bridge use, potentially indicating that transportation behaviors, after a bridge is installed, are no longer impacted by rainfall conditions. Higher bridge use was observed in the late afternoons, on market and church days, and roughly equal use of the bridge crossings in each direction. These trends are consistent with the design-intent of these bridges.

## 1 Introduction

Isolation caused by lack of transportation infrastructure makes access to basic social and economic activities unreliable for rural communities. This uncertain access to markets, income-generating opportunities, and health and education facilities contributes to persistent rural poverty [1]. The World Bank estimates that one billion people worldwide lack access to an all-weather road, illustrating the scope of the problem and the challenge of addressing it at scale [2].

**Fig 1:**
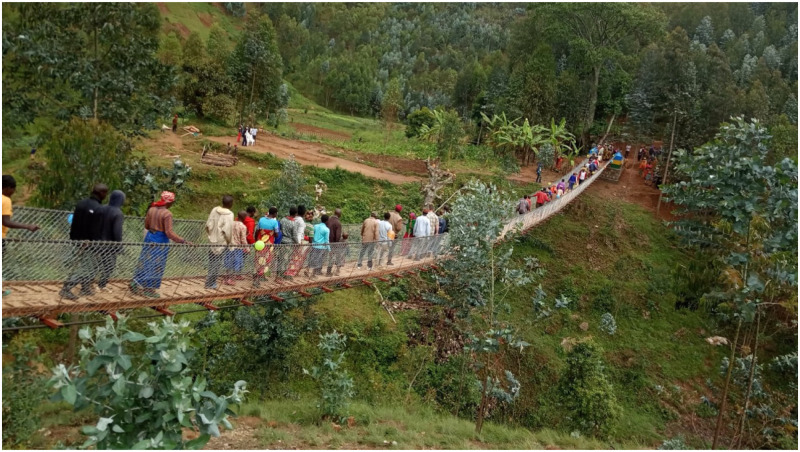
An example trail bridge constructed by Bridges to Prosperity in Rwanda.

Bridges to Prosperity (B2P) is a non-profit organization that builds trail bridges to connect isolated rural communities to road networks and critical destinations and services including markets, hospitals and schools. Fig 1 illustrates an example bridge location in Rwanda. B2P has constructed 339 trail bridges in 21 countries. A study in Nicaragua established economic and livelihood benefits attributable to these bridges [3].

In an effort to establish any economic, health or educational impacts of these trail bridges in Rwanda, we conducted a matched-cohort study over a 12 month period in 2018-2019. As part of this study, we installed motion-activated digital cameras at several of the bridge crossings. The images collected are intended to support characterizing bridge use. Objective measurement technologies and techniques are self-evidently important to develop and refine when supporting claims of the effectiveness of environmental interventions.

A variety of technologies and analysis methods have been deployed and validated to count pedestrians, bicycles and vehicles crossing bridges and other transportation infrastructure. These methods include in-person observational counting [4, 5], motion-activated counters [4], magnetic inductive loops [6], infrared light beams, pressure pads, thermal cameras [7], and digital video [8, 9] and imagery analysis [10].

These methods have been almost entirely deployed in high income urban settings, and often use technologies that would be cost-prohibitive or infeasible in a rural, low income setting. Bridges to Prosperity’s standard bridge use monitoring methods typically rely on manual, in-person data collection, which is time-consuming, produces temporally limited data, and is labor intensive. In order to identify and track trends and magnitude of bridge use, a continuous and automated method would be useful.

In this paper we describe the development, implementation, validation and findings of a novel method using low-cost, readily available motion-activated digital cameras in combination with open-source computer vision algorithms for measuring the use of these bridges. We describe the technology deployed, the computer vision supported detection algorithm applied, a human-validated error estimate, and early findings of bridge use patterns.

## 2 Methods

**Fig 2:**
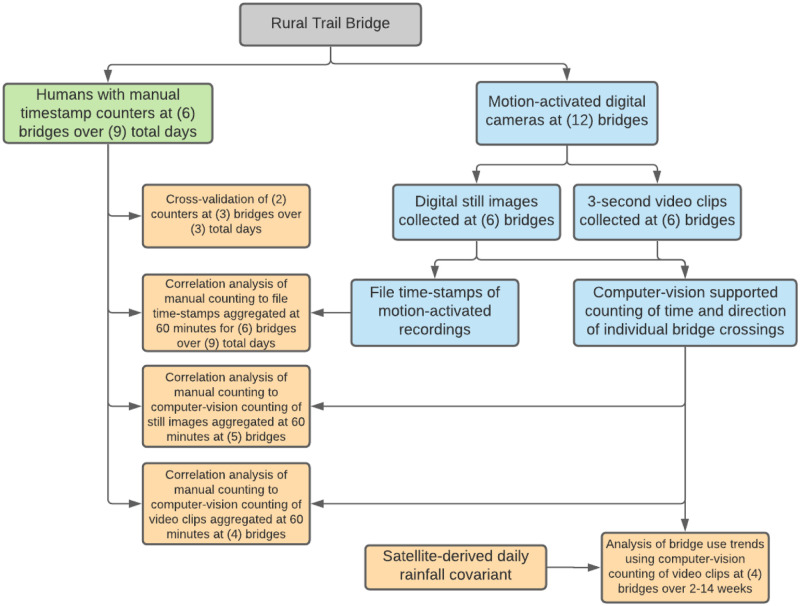
Flowchart describes the technologies and methods applied in this study. Digital cameras were installed at 12 total bridges, recording short video clips or digital still images. 60-minute aggregations of manual counting was then compared to the timestamps of the digital files from the cameras, and computer-vision supported counting of both the video clips and the digital stills. Following this validation, analysis of bridge use trends including satellite-detected rainfall as a co-variant was conducted for 4 bridges over 2-14 weeks in fall 2019. Green blocks indicate manual counting, blue blocks indicate digital data collection, and orange blocks indicate statistical analyses. Human manual counting was collected at several bridge sites in rural Rwanda, and cross-validated between two manual counters.

The following section details the technologies and methods deployed in this study. In brief, human manual counting was collected at several bridge sites and cross-validated between two manual counters. Digital cameras were installed at 12 total bridges, recording short video clips or digital still images. Manual counting was then compared to a.) the timestamps of the digital files from the cameras, b.) computer-vision supported counting of both the video clips and the digital stills. Following this validation, analysis of bridge use trends was conducted. Fig 2 presents a flowchart of these data collection technologies and analysis methods applied.

### 2.1 Camera selection, installation and image collection

**Fig 3:**
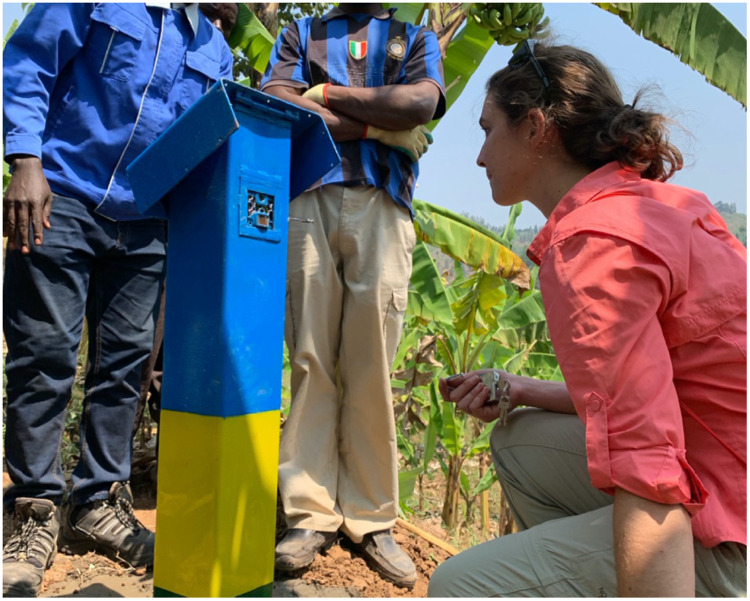
An example motion activated camera installed at a trail bridge in Rwanda in support of this study (co-author Gerster pictured).

In this study, we examined if a motion-activated digital camera system could support bridge use data collection. We reviewed several commercially available motion-activated digital cameras marketed for outdoor long term monitoring of wildlife. After comparing cost, complexity, battery lifetime and mechanical interfaces, we selected the Browning Spec Ops Advantage (Browning Trail Cameras, www.browningtrailcameras.com), available retail for about $150. Fig 3 shows one of these cameras installed in a protective housing next to a trail bridge entry ramp.

These cameras were installed at 12 bridge crossing sites over varying periods in 2019. Two modes of image data collection were employed—motion activated digital still images, and motion-activated short (3 second) video clips. The cameras automatically employed infrared LED lighting to support after-dark observations. Images were recorded on local data cards.

### 2.2 Manual counting

To support subsequent image analysis validation, at five sites we conducted daytime manual counting over 9 days, for about 8 hours per day. Counting was recorded with time-stamp electronic clickers. Each count represented one observed crossing of an individual, in either direction. Four of these day-long observations included two staff members independently and concurrently observing and recording bridge crossings in order to cross-validate this method.

### 2.3 Computer vision analysis

After varying periods of camera installation, the imagery files were recovered. For several sites, the still and video images were then “stitched” together to create continuous data files, then used to apply and refine computer vision algorithms for detecting and counting bridge users.

Computer-vision supported counting involves observing people in the video, tracking them as they move, and determining their direction of motion based on their tracks. We used modern deep neural networks and other machine vision tools provided by the open source OpenCV machine vision toolkit (www.opencv.org) to accomplish each of these steps automatically. The first step, finding people in an image, is an example of *object detection* [11], in which the goal is to find instances of specific types of objects and put bounding boxes around them. In this case, we used the open source Darknet [12] implementation of the YOLO (You Only Look Once) [13] object detection deep neural network that is pretrained to, among other things, detect people at frame rate.

Object detection has many applications, and thus has received significant attention in the machine vision community [11]. Popular approaches tend to yield algorithm “families”, such as R-CNN [14], Fast R-CNN [15], Faster R-CNN [16], and Mask R-CNN [17]. The latter learns to identify whether individual pixels belong to an object, a level of detail not needed for our application, while the first three place bounding boxes around detected objects. Later members of the R-CNN family tend to be faster, but not necessarily more accurate. The YOLO family includes YOLO, YOLO v2 [18], and YOLO v3 [19]. We chose YOLO due to its superior runtime performance over the R-CNN and the availability of pretrained models specifically for the person detection use case.

For all deep object detection methods, people are detected independently across frames. That is, the fact that a person is detected in frame *i* does not inform detections in frame *i* − 1 or frame *i* + 1. Tracks must be built from consecutive frames for each person. That is accomplished by some form of an *appearance model* that characterizes the visual appearance in each bounding box so that the matching bounding box, the one with the most similar appearance, can be found in subsequent frames. Common choices for tracking with appearance models are the DLIB correlation algorithm [20] and the Simple Online and Realtime Tracking with a Deep Association Metric (DeepSort) algorithm [21]. We used the latter as it integrated more easily with the rest of our system.

Note that errors can occur anywhere in the pipeline. While false positives are rare in the object detection stage, false negatives can and do occur, where people are missed in one or more frames due to lighting conditions, occlusion, debris on the camera lens, etc. Appearance models are often based on simple image descriptors, like color histograms, and can thus also lead to false or missed matches. That said, the empirical results, described below, suggest that the overall system is robust and accurate.

**Fig 4:**
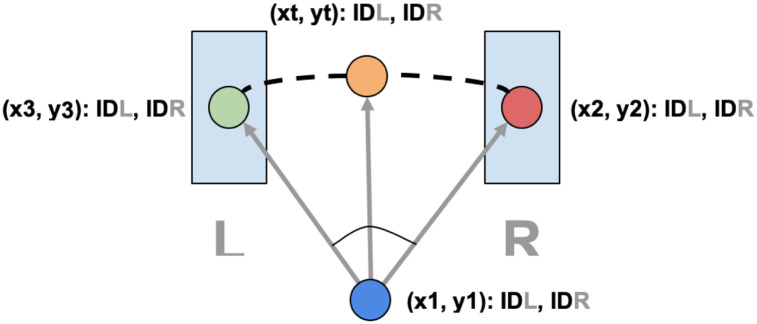
Deep learning people counter for left and right direction on a bridge. The angle that a tracked object makes with the line between two anchor points at the center of the bridge is tracked over time. The behavior of that value for a given detection ID indicates whether the traversal is left-to-right or right-to-left.

The geometric application of this algorithm is illustrated in Fig 4, wherein an object centroid is tracked moving from one side of the frame to the other (i.e, left to right).

We start by defining a distance threshold between each object and two anchors placed at (xt, yt) and (x1, y1). With these anchors, we can create unique lines and track the rotation they make with the trackable centroids at (x3, y3) and (x4, y4). When a subject enters right, the counter begins to count the angle defined by the points (xt, yt), (x1, y1) and (x2,y2). When a subject enters left, the counter begins to count the angle defined by the points (xt, yt), (x1, y1) and (x3, y3). Maintaining a unique id while counting is essential in this step. Each angle is added to its corresponding trackable object queue. Once an object exits the trackable line region, the sum of the difference between contiguous angles in their respective object queue will be positive or negative. Positive indicates left direction while negative indicates right.

### 2.4 Ethics—Human research subjects

At the sites where cameras are installed, it was not practical to secure informed consent from every person using the bridge. Instead, the cameras were installed in public locations, are highly visible, and include a placard in Kinyarwanda stating, “This camera was installed in (month, year) for research approved by the Rwanda National Ethics Committee. It is recording people crossing the bridge. This will help us to understand the impact the bridge is having on surrounding communities. Please do not damage it or try to steal it.” Further, our research protocol includes blurring the faces of people in any images or videos published. This statement and approach was approved by the Rwanda National Ethics Committee on January 28, 2019. The individual pictured in Fig 3 (co-author Gerster) has provided written informed consent (as outlined in PLOS consent form) to publish their image alongside the manuscript.

## 3 Results

### 3.1 Manual counting validation

**Fig 5:**
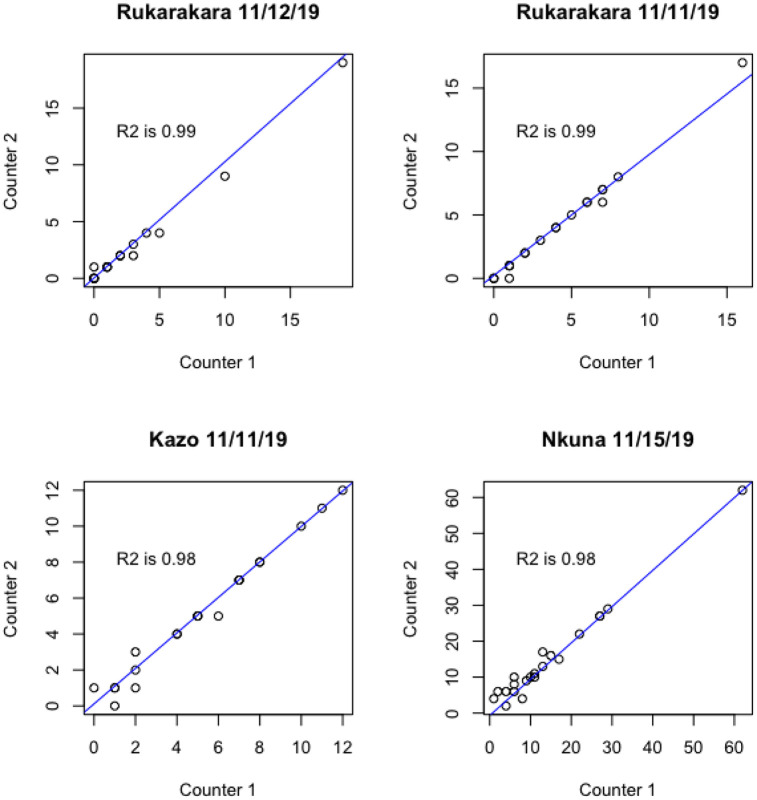
Scatterplots and linear regressions of two independent manual counters for four day-long observation periods at three bridge sites in November 2019. Counts are aggregated at 15 minute intervals. These results indicate nearly total agreement between the two manual counts (R^2^ > 0.98).

Manual counting by two separate staff were conducted across 4 days and 3 sites. A total of 1,713 separate crossings were observed by the manual counters. These two independent manual counts are compared to establish confidence in this method. Fig 5 illustrates a linear regression of each counter at each site, aggregated at 15 minute intervals. These results indicate nearly total agreement between the two manual counts (R^2^ > 0.98). Therefore, in subsequent analysis these manual counts are considered the ground-truth.

### 3.2 Motion activated event counting

**Fig 6:**
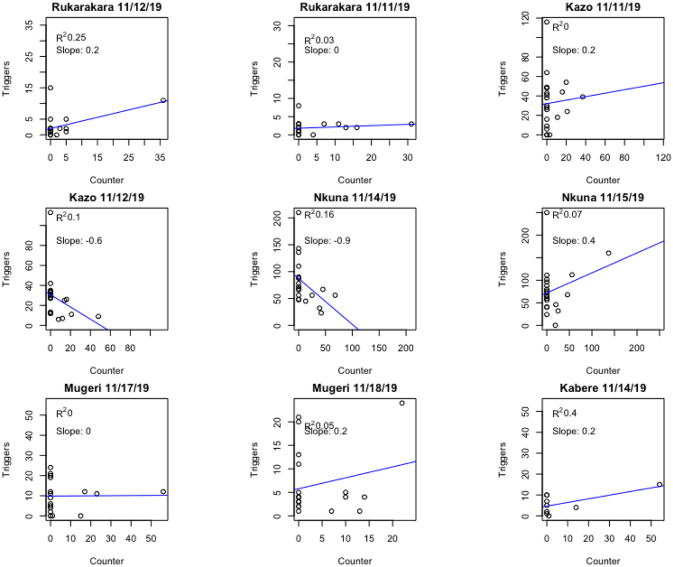
Scatterplots and linear regressions of manual counting versus motion-activated timestamps of image files for nine day-long observation periods at six bridge sites in November 2019. Manual and digital file timestamps are aggregated at 60 minute intervals. Poor correlations (R^2^ range 0–0.4) between these counting methods are observed across all tested sites, suggesting that motion-detector based counting is not a reliable indicator of bridge crossings.

In previous work, we and others have relied on motion-activated event counting applied to the use of sanitation infrastructure [22–24]. The digital cameras used in this study create separate files for each motion-activated image or video. We used these time-stamped image files to establish if simple event detection similar to the latrine monitors deployed in other studies (without image analysis) could be a sufficient measure of bridge use. Fig 6 shows manual counting compared to motion-activated timestamp events (digital files) aggregated at 60 minute intervals for 9 day-long observation periods at 6 bridge sites. As illustrated, there are poor correlations between these counting methods across all tested sites. This poor correlation suggests that motion-detector based counting would not be a reliable indicator of the total number of bridge crossing events.

### 3.3 Computer vision supported counting

**Fig 7:**
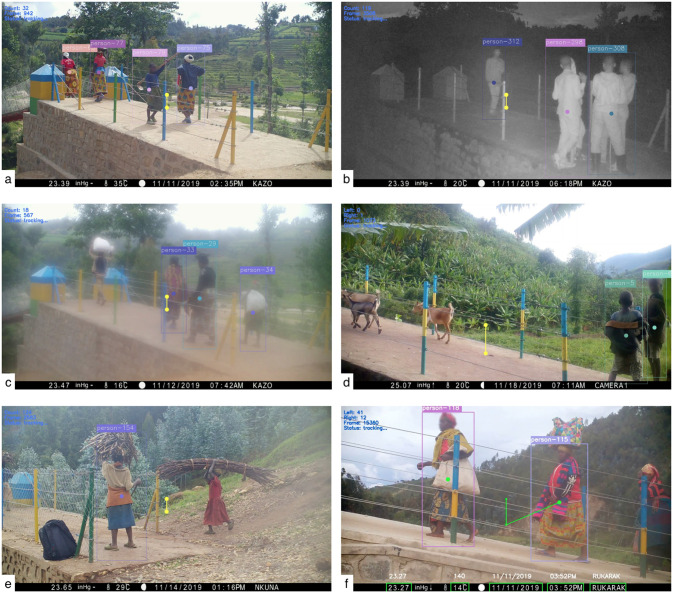
Example camera frames and computer vision supported people counting at four bridge crossings in Rwanda. Faces have been blurred to protect identities consistent with research protocols. In (a), We see four people are identified crossing the centerline to the left. In (b) we see the same site at night, where the infrared illumination is sufficient to capture individuals, including distinguishing those socializing versus crossing the bridge. In (c), observe the same site in the morning while condensation is apparent on the camera lens. The algorithm is still able to identify and count individuals. In (d), another site where people are identified and counted while animals are not. In (e) and (f) two other example bridges with varying camera locations and angles, and subjects.

Adapting the OpenCV computer vision people counter described above, we analyzed videos and photos collected at these 6 sites over 9 day-long observation periods. Fig 7 presents six example screen shots from this analysis, representing a range of camera installation positions, bridge crossing behaviors, weather conditions, and lighting conditions. In each case, individuals are identified and counted when they cross the center of the frame.

**Fig 8:**
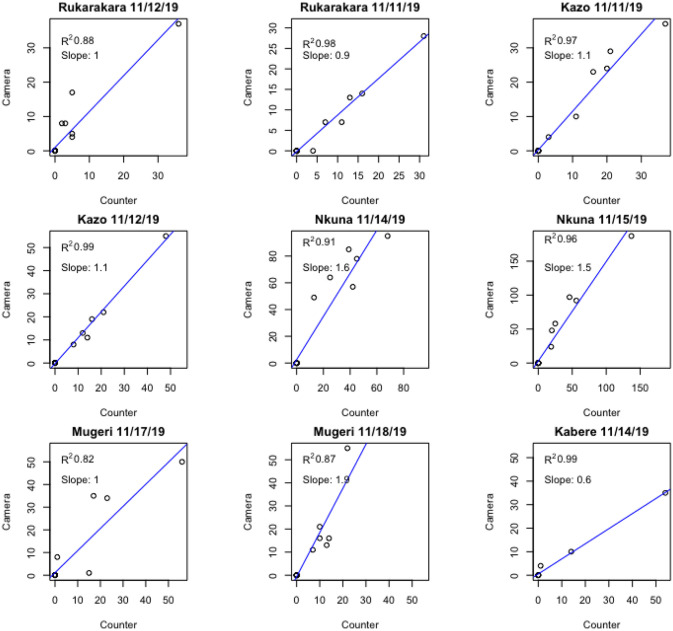
Scatterplots and linear regressions of manual counting versus computer vision supported counting of both video-clip and digital-stills for nine observation periods at six bridge sites in November 2019. Manual and computer counts are aggregated at 60 minute intervals. Strong correlations between manual counting and computer-vision counting are observed (R^2^ range 0.82–0.99).

**Fig 9:**
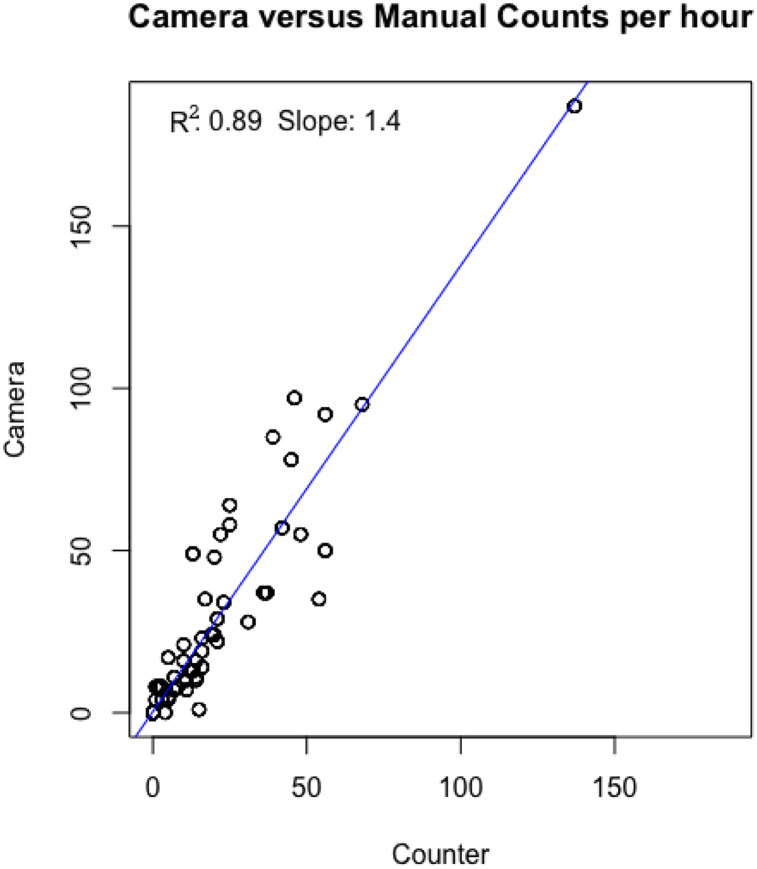
Scatterplot and linear regression of aggregated manual counting versus computer vision supported counting of both video-clip and digital-stills across nine observation periods at six bridge sites in November 2019. Manual and computer counts are aggregated at 60 minute intervals and show strong overall correlation (R^2^ = 0.89).

Fig 8 shows manual counting compared to computer vision supported counting aggregated at 60 minute intervals for 9 day-long observation periods at 6 bridge sites, while Fig 9 reflects this same data aggregated across sites.

As illustrated, there is some variability in correlation between the computer vision counts and the manual counts between sites. We found that the motion-activated video-clip files provided greater support for the computer vision algorithm compared to the stills. Table 1 presents error estimates disaggregating by the video and photo-still data types. The overall error bias of the video-clip data type was 2.63% per hour of counting.

**Table 1:** Computer-vision supported hourly aggregated counting of pedestrian crossings using motion-activated short videos and photographic stills compared to manual in-person counting. Error estimates for variance and bias indicate a lower error using the videos compared to stills.

Parameter	Variance	Bias
Video-supported Error	25.05%	2.63%
Sites	4	
Manual Counts	319	
Still Photo-supported Error	53.66%	50.06%
Sites	5	
Manual Counts	838	

### 3.4 Bridge use trends

**Fig 10:**
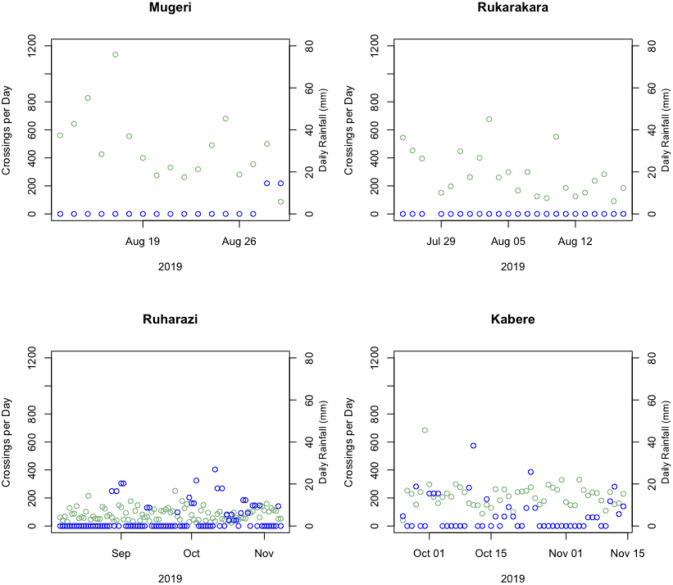
Daily total bridge crossings detected with computer vision algorithm, and daily CHIRPS remotely observed rainfall for four bridge crossings over about 2-14 weeks in fall 2019. Non-zero rainfall is observed only for the Ruharazi and Kabere bridge sites, for which observations were available during the rainfall season September-November. Site-level and aggregated linear regressions (not shown) of daily bridge crossings compared to daily rainfall or 7-day mean rainfall did not indicate any correlation, indicating that bridge use during these observation periods at these sites was not dependent on rainfall.

Based on the findings that our computer vision algorithm supported by the motion-activated video clips has a low error bias per hour of counting, we then conducted computer vision people counting for the 4 sites for which we had video-clip data types across longer observational periods ranging between 17 and 51 days of continuous observation during August—November 2019. Fig 10 illustrates these estimated total daily crossings, along with daily rainfall at these sites. Rainfall estimates are provided using the remote-sensing based Climate Hazards Group InfraRed Precipitation with Station data (CHIRPS) [25]. Table 2 presents statistics on the observed crossings at these four sites.

**Table 2:** Computer vision algorithm counted bridge crossings using motion-activated video clips at four sites over about 2-14 weeks in fall 2019. Average daily bridge crossings range 85-478, daily standard deviations range 45-249.

Site /Parameter	Kabere	Rukarakara	Ruharazi	Mugeri
Daily Crossings				
Mean	478	287	85	218
Standard deviation	249	161	45	91
Minimum	87	91	23	41
Maximum	1138	676	251	684
Sample (days)	17	23	95	51

Site-level and aggregated linear regressions of daily bridge crossings compared to daily rainfall or 7-day mean rainfall did not indicate any correlation, indicating that bridge use during these observation periods at these sites was not dependent on rainfall. As these observation periods captured only part of the year, we sought to establish if the rainfall variability and extremes observed during this period were representative of likely rainfall patterns throughout the year. Table 3 presents the observed rainfall mean, standard deviation, minimums and maximums recorded for each site during the observation periods, and for July 2017 to June 2018. An unpaired t-test of the total sample for these four sites of the rainfall during the observation period and over a 3-year period indicated no significant difference, suggesting that the observational period may be sufficient in capturing typical rainfall variability and any subsequent attribution of rainfall to bridge use.

**Table 3:** Rainfall variability during camera observation periods and over 2017-2020. Unpaired t-test comparing 3-year rainfall variability to rainfall observed during camera-observation period in 2019 indicated no significant difference, suggesting the observational period captures typical rainfall variability and any subsequent attribution of rainfall to bridge use.

Site /Parameter	Kabere	Rukarakara	Ruharazi	Mugeri
Daily rainfall -observation period (mm)				
Mean	1.71	0	3.28	5.15
Standard deviation	4.84	0	6.22	8.14
Minimum	0	0	0	0
Maximum	14.57	0	26.91	38.25
Mean daily rainfall -July 2017—June 2020 (mm)				
Mean	4.56	4.65	4.14	3.27
Standard deviation	8.11	8.43	7.76	5.73
Minimum	0	0	0	0
Maximum	64.47	78.71	77.74	49.73

**Fig 11:**
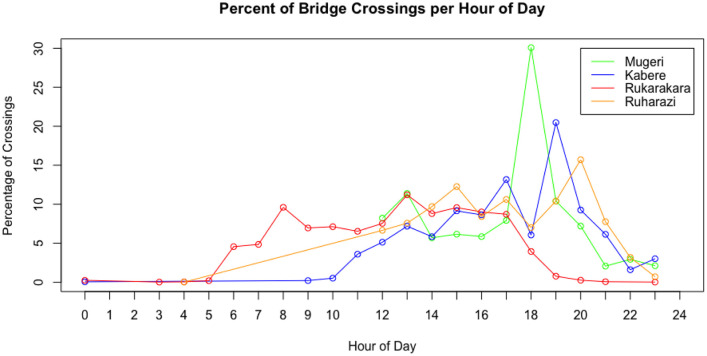
Percentage of bridge crossings per hour of day for four bridge sites over the observation period in fall 2019. Trends indicate high late afternoon use.

**Fig 12:**
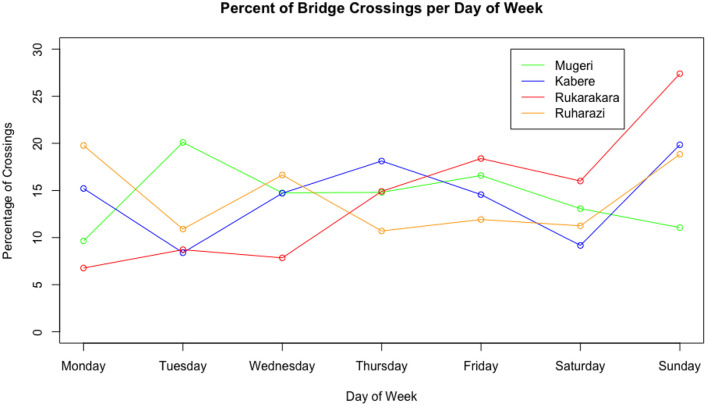
Percentage of bridge crossings per day of week for four bridge sites over the observation period in fall 2019. Hourly use trends indicate higher use on Sundays, which are market and church days in these communities.

**Fig 13:**
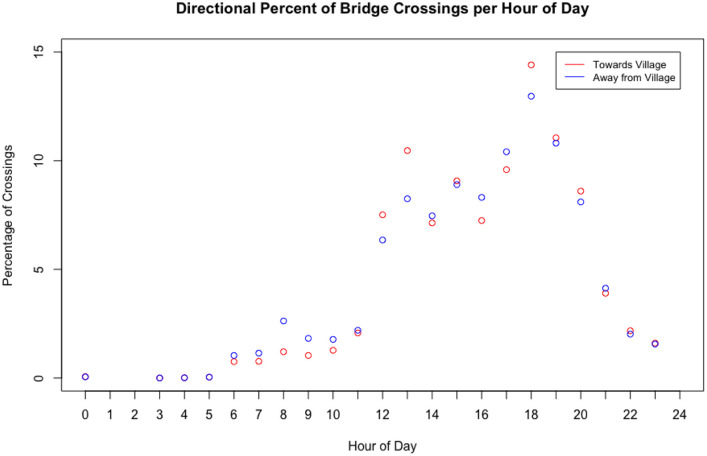
Percentage of bridge crossings in each direction for each hour of the day for four bridge sites over the observation period in fall 2019. These trends indicate roughly equal total use of the bridge crossings in each direction, suggesting that individuals return across the bridge.

We then examined site-level and aggregate bridge use trends. Fig 11 shows the percentage of bridge crossings per hour of day for each site over the observation period. The trends indicated in the plot suggest high late afternoon use at all sites. Fig 12 shows the percentage of bridge crossings for each day of the week at each site. These trends indicate higher use on Sundays, which are market and church days in these communities. Finally, Fig 13 shows the percentage of bridge crossings in each direction for each hour of the day. “Towards Village” indicates individuals crossing the bridge in the direction of the community identified as most-impacted by the bridge, while “Away from Village” indicates individuals traveling out of the community. These trends indicate roughly equal use of the bridge crossings in each direction.

## 4 Discussion and forward work

This study developed and validated an accurate and useful method for counting and characterizing the use of trail bridges in rural Rwanda. In this study, we deployed motion-activated digital cameras across several trail bridge sites in Rwanda. We conducted and validated manual counting of bridge use to establish a ground truth. We adapted an open source computer vision algorithm to identify and count bridge use reflected in the digital images. We found a reliable correlation with low mean error of bridge crossings per hour between manual counting and those sites at which the digital cameras collected short video clips when triggered.

We then applied this algorithm across 186 total days of observation at four sites in fall 2019, and observed a total of 33,800 daily bridge crossings ranging from about 20 to over 1,100 individual uses per day, with no apparent correlation between daily or weekly rainfall and bridge use. Bridge use trends were consistent with the design-intent of these bridges indicating higher use on market and church days, and roughly equal use of the bridge crossings in each direction.

Bridges to Prosperity’s theory of change posits that rural communities are periodically and dangerously isolated by flooding events, and that trail bridges eliminate this isolation and risk. The analysis presented in this paper suggests that bridge use is not dependent on rainfall, potentially indicating that communities prefer the trail bridges to alternative or baseline river crossings. However, while no rainfall dependence on bridge use was observed, further investigation is required to establish if there are any seasonal attributes to bridge use (such as harvest) or extreme weather events (flooding).

The work presented in this paper was conducted in support of a large scale (approximately 200 site) randomized controlled trial currently being conducted and scheduled for completion in 2024. As part of the large-scale study, we plan to deploy about 50 of these camera systems at bridge sites. The findings presented in this paper suggest that between-site variability in bridge use may be more significant than within-site variability. This may motivate moving camera systems between sites. Further, this large scale study will provide an opportunity to compare bridge use patterns to community level economic, health and educational outcomes.

The computer vision algorithm we deployed also detected the direction (left-to-right and right-to-left) movement of the subjects. Additionally, as illustrated in the example images provided above, the nature of bridge use can be in part deduced through review of the collected images. The cameras also record local ambient temperature and barometric pressure. These additional data and capabilities may support further opportunities for bridge use characterization and modeling.

## Supporting information

S1 File (ZIP)
